# How do low-complexity domain interactions control transcription?

**DOI:** 10.1042/BST20250199

**Published:** 2026-10-07

**Authors:** Jiamin Guo, Qinyu Han, Kyogo Nagashima, Shasha Chong

**Affiliations:** Division of Chemistry and Chemical Engineering, California Institute of Technology, Pasadena, CA 91125, U.S.A.

**Keywords:** intrinsically disordered regions, Low-complexity domains, phase separation, transcriptional regulation

## Abstract

A substantial portion of the proteome consists of intrinsically disordered regions (IDRs) that do not fold into well-defined structures. IDRs often comprise low-complexity domains (LCDs), whose repetitive, compositionally biased sequences drive multivalent interactions that can lead to liquid–liquid phase separation, forming biomolecular assemblies. These LCD-mediated assemblies play an important role in transcriptional control, and productive output depends not on their mere presence but on how strongly and selectively LCDs interact. The present review integrates the optimal-interaction, or ‘Goldilocks,’ principle with RNA polymerase II phosphorylation and RNA-mediated feedback into a unified mechanistic framework spanning the transcription cycle. We connect dysregulation of LCD-mediated multivalent interactions to pathological transcription in cancer, neurodegeneration, and developmental disorders, whether through oncogenic LCD fusions, disease-associated post-translational modifications, or mutations altering phase-separation propensity. We further summarize experimental approaches for studying LCD function, discuss current methodological limitations, and highlight directions for future research.

## Introduction

Whereas many protein domains adopt well-defined three-dimensional structures, intrinsically disordered regions (IDRs) instead exist as dynamic conformational ensembles that are challenging to interrogate using classical structural biology approaches [1,2]. IDRs are increasingly recognized as important regulators of cellular organization and gene expression, and their dysregulation is implicated in diverse diseases, including cancer, neurodegeneration, and developmental disorders. Many IDRs comprise low-complexity domains (LCDs), which are enriched in repetitive or compositionally biased amino acid motifs [3–5]. Not all repetitive sequences are disordered, however: structured proteins such as elastin, silk fibroin, and collagen also contain repetitive motifs, but these instead drive the formation of stable fibrillar assemblies [6–8]. The present review focuses on intrinsically disordered LCDs rather than such folded repetitive domains.

Many LCDs engage in multivalent protein–protein interactions that drive the formation of biomolecular assemblies involved in transcriptional regulation, chromatin remodeling, and RNA metabolism [9–11]. These assemblies feature heterogeneous composition and material properties, which can change with cellular conditions [1,12]. Such structures have been described using terms including ‘hubs,’ ‘condensates,’ ‘droplets,’ and ‘puncta.’ The term ‘condensate’ was initially linked specifically to liquid–liquid phase separation (LLPS), but its definition has since broadened to any membrane-less compartment that selectively concentrates biomolecules, regardless of whether LLPS is involved [1,12,13]. We adopt this broader definition and use ‘assemblies’ as a neutral, inclusive term, while preserving terms where they reflect the original studies’ usage.

LCDs within transcription factors (TFs), coactivators, and RNA polymerase II (RNAP II) organize transcriptional machinery into dynamic nuclear assemblies that concentrate regulatory factors and enable gene transcription [9–11,14,15]. Transcriptional output is dependent on both the formation and regulation of such assemblies [1,16–18]. The present review discusses the theme across four related topics: sequence-encoded interaction rules, RNAP II assembly formation and regulation, the Goldilocks behaviors underlying optimal transcriptional output, and disease-associated dysregulation.

Unlike our recent methodological review on single-molecule imaging of transcription-regulating IDRs [19], the present review focuses on the biological and mechanistic questions of how LCD sequence features and interaction networks control transcription in physiology and disease. It is further distinguished from other recent reviews on transcriptional condensates that survey condensate biology broadly [20] or focus on RNAP II compartmentalization [21]. Rather than treating LCD–LCD interactions, RNA-mediated feedback, and RNAP II LCD phosphorylation as separate themes, we integrate them into a single mechanistic model spanning the transcription cycle and connect this framework directly to disease.

## Sequence features of LCDs and regulation of their assemblies

LCDs are enriched in compositionally biased polar, aromatic, and charged residues arranged in repetitive or patterned motifs [13,22]. Rather than adopting stable secondary or tertiary structures, these sequences enable dynamic, multivalent interactions whose strength, reversibility, and selectivity are highly sensitive to this composition and patterning [13,22–24]. The sticker-spacer model explains how residue types contribute to assembly formation (Figure 1A) [13,22]. Aromatic residues, such as tyrosine and phenylalanine, act as ‘stickers’ that drive weak π–π stacking or π–cation interactions, while intervening polar residues, such as serine, glutamine, and glycine, act as flexible ‘spacers’ that keep these interactions weak and transient. This sticker-spacer balance enables dynamic, reversible assemblies that interact with diverse cellular partners in adaptable conformations. Proteome-wide analyses of IDRs across thousands of eukaryotic proteins, which include many LCDs, have revealed that aromatic content and spacing, charge distribution, and sticker density influence multivalent interactions and predict phase-separation propensity [23,25].

**Figure 1:**
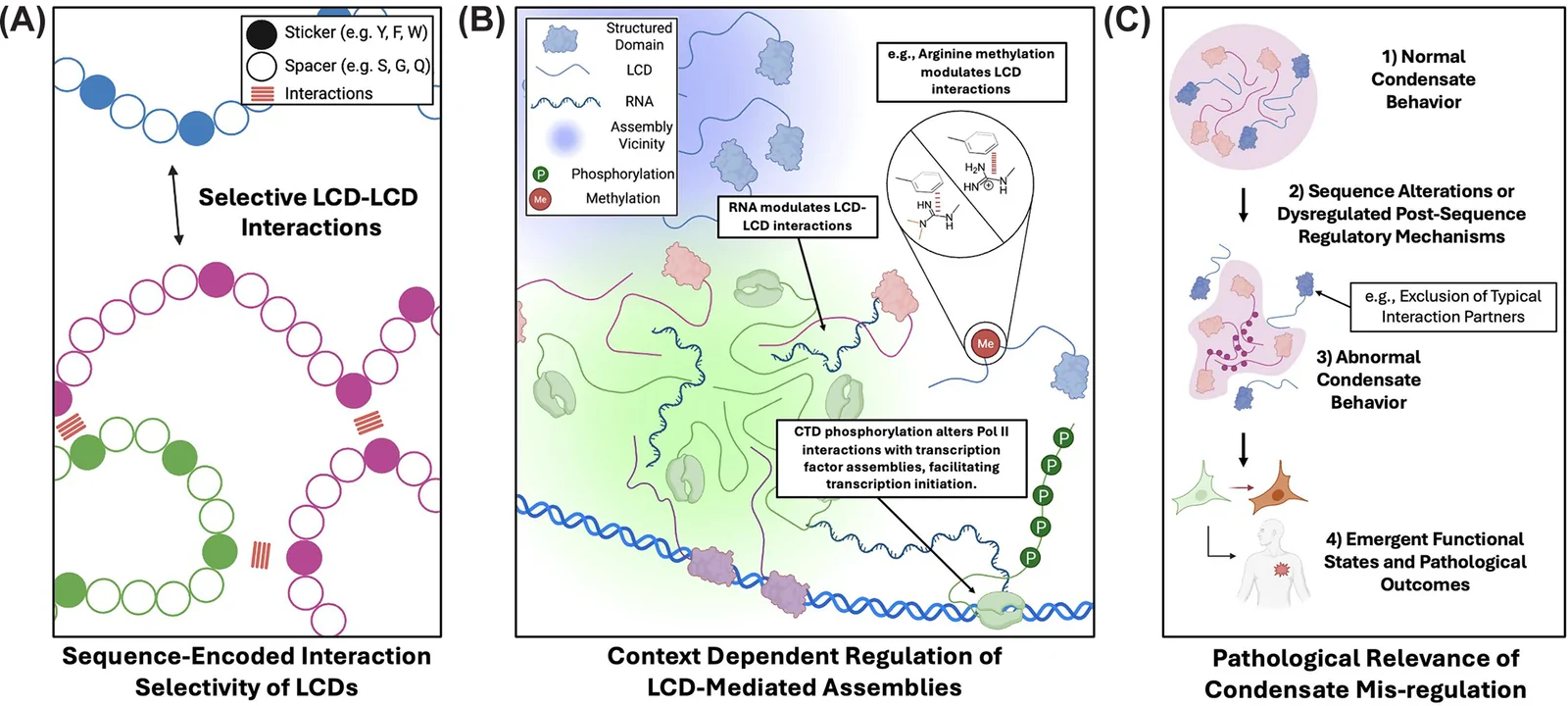
Multiscale control of biomolecular assembly formation, function, and pathological outcomes (**A**) Sequence-encoded interaction selectivity of LCDs. Despite lacking a stable tertiary structure, LCDs encode selective interaction propensities through their amino acid composition and patterning. In the sticker-spacer model, recurrent ‘sticker’ residues embedded in flexible spacer regions mediate specific LCD–LCD interactions that underlie assembly formation and its biophysical properties. (**B**) Context-dependent regulation of transcription assemblies. Beyond intrinsic sequence features of LCDs, assembly behavior is further shaped by the cellular environment. Post-translational modifications (PTMs), RNA binding, and interactions with structured protein domains modulate LCD–LCD interactions, influencing interaction partner recruitment and molecular exchange in assemblies. In transcriptional assemblies, regulatory inputs, including RNA-mediated modulation and phosphorylation of the RNAP II CTD, reshape interaction networks of TF assemblies and are associated with changes in transcriptional states. (**C**) Pathological relevance of assembly misregulation. Disruption of sequence-encoded interactions or their regulatory control of assemblies—arising from mutations, aberrant PTMs, or altered cellular context—can lead to abnormal assembly composition and dynamics, giving rise to emergent functional states and pathological outcomes.

PTMs are known to modulate LCD behaviors and the material properties of their assemblies (Figure 1B) [26]. For example, tyrosine phosphorylation of the LCD of hnRNPA2, an RNA-binding protein that forms neuronal mRNA transport granules, reduces its propensity to phase separate relative to the unmodified protein and suppresses the aggregation of disease-associated hnRNPA2 variants, whose LCD mutations otherwise drive an irreversible transition from dynamic condensates into solid aggregates [27]. Similarly, O-GlcNAcylation of the EWSR1 LCD reduces its propensity for phase separation and increases the fluidity of its condensates, preventing irreversible maturation [28].

## LCD-mediated interactions in transcriptional control

### Adaptable and selective LCD–LCD interactions

Unlike structured domains that mediate specific interactions through rigid, fixed-stoichiometry interfaces, LCDs engage in multivalent, transient interactions whose effective stoichiometry scales with local LCD concentration. This allows LCD-containing proteins to participate in diverse regulatory processes. The adaptability is exemplified by the FET family proteins (FUS, EWSR1, and TAF15), whose LCDs self-interact and engage in heterotypic multivalent interactions with partners involved in transcription, RNA processing, and stress responses. For example, FUS and EWSR1 LCDs interact with multiple components of the transcriptional machinery, including RNAP II and chromatin remodelers such as the mSWI/SNF complex [29–31]. These interactions allow FET LCDs to redistribute across distinct nuclear assemblies under different conditions [32–34].

Despite this versatility, LCD interactions are highly selective. FET family LCDs self- and cross-interact but do not interact with the LCD of Sp1 [9]. LCDs within TAF15, EWSR1, and Sp1 each nucleate phase-separated condensates of distinct protein composition, reflecting divergent multivalent interactomes despite that all three LCDs function as transactivation domains [35].

TF IDRs also exhibit regulatory behaviors in addition to driving multivalent interactions. Although they do not bind to DNA directly, IDRs direct TF binding to most target promoters *in vivo*. They function as signal integrators, combining short linear motifs (SLiMs) with SLiM-independent sequence composition grammars to enable context-dependent genomic targeting [36,37]. They also accelerate TF’s target search through condensate incorporation and nonspecific chromatin contacts [38]. TF IDRs further mediate transcriptional activity and specificity through their residue composition: hydrophobic residues confer transcriptional activity, acidic residues prevent autoinhibition by counteracting nonspecific intramolecular folding of the IDR onto the TF’s DNA-binding domain, and hydrophilic residues tune genomic binding specificity [39]. Consistent with this, the balance between acidic and hydrophobic residue clusters governs transactivation potency; sequence-based clustering models can further predict transactivation domains across human TFs [40–42].

LCD-mediated regulation also influences chromatin-scale processes. For example, heterochromatin protein 1 (HP1) family members contain IDRs that contribute to multivalent interactions and, particularly for HP1α, can promote LLPS. HP1-mediated condensation has been proposed to compact H3K9me2/3-marked chromatin into heterochromatic domains, a process regulated by phosphorylation of the HP1α N-terminal IDR and multivalent interactions with methylated histones [43,44].

### LCD-mediated assembly of transcriptional machinery: a focus on RNAP II

RNAP II recruitment, activation, and progression are coordinated by LCDs that organize transcriptional machinery into transient nuclear assemblies (Figure 1B) [9–11], enabling dynamic recruitment and release of regulators for precise temporal control. Central to this process is the RNAP II C-terminal domain (CTD), an LCD of 52 tandem heptapeptide repeats (Tyr-Ser-Pro-Thr-Ser-Pro-Ser) whose phosphorylation-sensitive interactions reconfigure transcriptional assemblies throughout the transcription cycle [15,45–47] (Figure 2).

**Figure 2:**
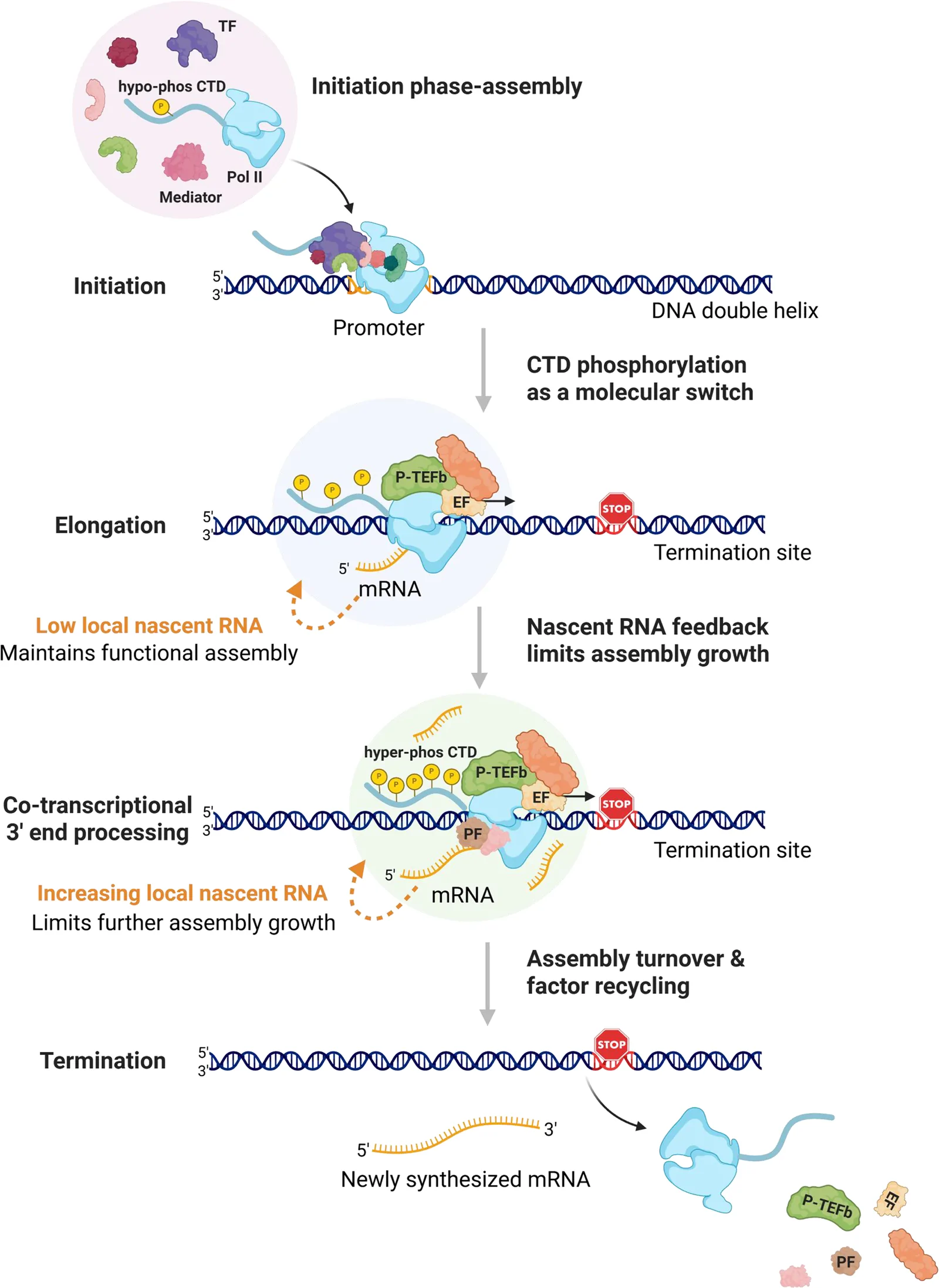
LCD-mediated interactions coordinate dynamic transcriptional assemblies throughout the RNA polymerase II transcription cycle At transcription initiation, multivalent interactions involving LCDs in TFs and coactivators, together with the hypophosphorylated CTD of RNA polymerase II (Pol II), concentrate transcriptional machinery at the promoter and support formation of an initiation-phase assembly. CTD phosphorylation subsequently acts as a molecular switch that reconfigures the CTD interaction network and facilitates the transition into productive elongation. At relatively low local levels of nascent RNA, functional transcriptional assemblies can be maintained, whereas increasing nascent RNA provides negative feedback that limits further assembly growth and favors assembly turnover. During late elongation, the hyperphosphorylated CTD associates with LCD-rich 3′-end RNA-processing factors, thereby coupling transcription to 3′-end RNA processing. Following termination, Pol II and associated factors dissociate and are recycled, while the newly synthesized mRNA is released. The RNA feedback depicted here reflects the concentration-dependent, growth-limiting relationship established for FUS, Mediator, and related transcriptional assemblies; RNA can act as a reinforcing rather than limiting cofactor in specific disease contexts, as discussed in the main text. The assemblies shown here represent dynamic multivalent interaction networks and do not necessarily imply stable phase-separated compartments. (CTD, C-terminal domain; EF, elongation factor; LCD, low-complexity domain; PF, 3′-end RNA-processing factor; Pol II, RNA polymerase II; P-TEFb, positive transcription elongation factor b; TF, transcription factor).

In its hypophosphorylated state, the CTD engages in multivalent interactions that nucleate initiation-phase assemblies of TFs, coactivators, and chromatin remodelers [11,12,15]. TFIIH kinase CDK7-mediated CTD Ser5 phosphorylation facilitates promoter escape of RNAP II, and CTD Ser2 phosphorylation by the elongation kinase complex P-TEFb drives RNAP II release into productive elongation [48–52]. As transcription proceeds along the gene body, increasing Ser2 and decreasing Ser5 phosphorylation further remodels the CTD interactome, stabilizing the elongation complex and facilitating eventual disengagement of RNAP II from DNA at transcription termination [47].

This phosphorylation-driven switch has its roots in the sequence-encoded phase-separation behavior of the CTD. Tyrosine aromaticity and *cis*-trans proline isomerization are the primary determinants of CTD phase separation, with SPXX motif prolines adopting β-turn conformations that compact the CTD ensemble and enhance inter-repeat contacts [53]. This phase-separated state, in turn, accelerates its own phosphorylation: CDK7 associates with the surface of CTD condensates, where it phosphorylates CTD far faster than it does in the dispersed state, enhancing phosphorylation efficiency during transcription initiation [53]. Phosphorylation not only dissolves CTD condensates but also actively reconfigures them, with phosphorylated CTD selectively associating with distinct regulators to form new types of condensates [54]. This is consistent with earlier evidence that hypophosphorylated CTD partitions into Mediator condensates during transcription initiation, while hyperphosphorylated CTD instead associates with splicing-factor condensates [55].

Transcription elongation also involves phase separation of other proteins besides RNAP II CTD. NDF, an IDR-containing factor that destabilizes nucleosomes, forms condensates with the histone chaperone FACT. These condensates travel with elongating RNAP II, promoting nucleosome disassembly while retaining histones. Disrupting NDF-FACT phase separation reduces FACT chromatin occupancy and impairs transcription elongation [56]. Phase separation is similarly implicated in 3′ end processing at the end of the transcription cycle. CPSF6, a component of the mammalian cleavage factor I complex, undergoes LLPS, and increased CPSF6 condensation is associated with preferential use of distal poly(A) sites; CLK2-mediated phosphorylation of its arginine/serine-like domain disrupts this condensation [57].

### The Goldilocks principle

Productive transcription has been proposed to depend on an optimal range of LCD-mediated interaction levels, referred to as the ‘Goldilocks’ principle [9,17,58]. Transcriptional output is a non-monotonic function of multivalent interaction levels: insufficient interactions fail to nucleate productive assemblies, while excessive interactions drive aberrant condensation that impairs transcription, possibly by sequestering regulators.

The deleterious consequences of excessive multivalent interactions are exemplified by the fusion TF EWS::FLI1 that contains an EWSR1-derived LCD and is known to drive the oncogenic transcriptional program in Ewing sarcoma. We observed that increased LCD-mediated condensation of EWS::FLI1 at target gene loci represses transcription [16]. Similarly, the androgen receptor requires an optimal level of its LCD-mediated interactions to drive enhancer assembly and coactivator recruitment; both weakened and excessive interactions impair enhancer assembly and reduce transcriptional output [58]. Likewise, cancer-associated mutants of ENL, a histone-acetylation reader, form small, functional, chromatin-associated condensates at near-endogenous levels, but marked overexpression of the ENL mutants drives large, chromatin-excluded, non-functional condensates [59].

RNA-mediated feedback provides an additional layer of regulation that helps maintain transcriptional assemblies in states conducive to optimal transcriptional output (Figure 1B). RNA tunes the multivalent LCD–LCD interactions of RNA-binding proteins such as FUS in a concentration-dependent manner: low-to-moderate RNA levels are permissive for condensate formation and co-partitioning with the RNAP II CTD, whereas higher levels weaken protein–protein interactions through competition and charge screening [17,60]. Similar RNA-dependent modulation has been observed for transcriptional coactivators Mediator/BRD4 at active enhancers [17]. The degree and direction of RNA regulation, however, depend on the condensate-forming protein and its underlying interaction chemistry. Depleting nascent RNA increases the chromatin-binding stability of oncogenic EWS::FLI1 hubs without altering their dimensions, unlike Mediator condensates, whose size and lifetime both increase with transcriptional inhibition [17]; this difference likely reflects the distinct molecular basis of EWS::FLI1 multivalent interactions, which depend on aromatic residues rather than the RNA-sensitive charge patterns that drive condensation of the Mediator subunit MED1 [17,61]. Conversely, for cancer-associated ENL mutants, locally produced RNA binds a conserved basic patch within the histone-reader YEATS domain, thereby actively reinforcing ENL condensate nucleation and chromatin engagement. Disrupting this ENL–RNA interaction impairs oncogenic transcription and leukemogenesis *in vivo* [62]. Thus, RNA can act as either a destabilizing or reinforcing regulator of condensates, depending on the molecular context.

### LLPS versus multivalency: an open question

A fundamental and actively investigated question is whether phase-separated condensate formation *per se* is required for transcriptional activation, as proposed in early condensate models of transcriptional control [10,11], or whether the underlying multivalent interactions are themselves sufficient. TF activation domains, which frequently contain IDRs, mediate multivalent interactions that alone enhance transcription by extending chromatin residence time and facilitating coactivator recruitment, with transcription activity better correlated with interaction kinetics than with the presence of condensates *per se* [63,64]. A direct comparison of soluble-complex and condensate models using the yeast TF Gcn4 showed that its propensity to form soluble complexes with the coactivator Med15 closely mirrored its propensity to recruit Med15 into condensates. These two properties are therefore deeply intertwined and difficult to disentangle experimentally, making it challenging to determine which is primarily responsible for Gcn4 function [65]. Notably, Gcn4 variants with the highest affinities for Med15 exhibited lower transcriptional activities than predicted by their binding affinities, with their activities attenuated by phase separation with Med15, underscoring that excessive heterotypic interactions within condensates can be counterproductive [65]. Rather than resolving whether LLPS is strictly required, these findings collectively point to multivalent interaction levels and kinetics as factors that contribute to transcriptional control.

### A unified model

Despite these open questions, LCD-mediated multivalent interactions, RNA-mediated feedback, and RNAP II CTD phosphorylation dynamics can be integrated into a unified model of transcriptional control (Figure 2). At transcription initiation, the hypophosphorylated CTD nucleates assemblies with Mediator, TFs, and coactivators within the Goldilocks window. CDK7-dependent Ser5 phosphorylation then shifts RNAP II toward transcription elongation, while accumulating nascent RNA typically weakens residual LCD-mediated interactions to help maintain a transcriptionally productive assembly. Subsequent Ser2 phosphorylation further remodels the CTD interactome, stabilizing the elongation assembly and priming RNAP II's transition to termination-associated machinery. Perturbation of any of these regulatory axes—through oncogenic LCD fusions, disease-associated PTM changes, or point mutations that alter phase-separation propensity—can shift the system outside its productive range, resulting in transcriptional dysregulation and disease.

## Pathological transcription driven by LCD–LCD interactions

LCD-mediated interactions are central to normal transcriptional regulation, and their dysregulation distorts transcriptional assemblies and drives pathological states across a wide range of human diseases (Figure 1C).

In cancer, many oncogenic fusion proteins repurpose LCDs for transcriptional reprogramming. In Ewing sarcoma, the LCD of EWS::FLI1 drives its aberrant co-condensation with mSWI/SNF chromatin remodelers, disrupting transcriptional homeostasis [66]. In acute myeloid leukemia, several NUP98 fusion proteins, such as NUP98::HOXA9, exploit their LCDs to aberrantly enrich transcriptional machinery at leukemogenic gene loci [67,68]; likewise, cytoplasmic mutant nucleophosmin (NPM1c) sustains pathogenic RNAP II recruitment at leukemogenic genes, including the *HOXA/B* cluster and *MEIS1* [69,70]. Similarly, in acute lymphoblastic leukemia, the MEF2D::hnRNPH1 fusion protein uses the hnRNPH1 LCD to aberrantly activate leukemogenic transcription [71,72]. These diverse oncogenic mutations converge on a shared mechanism across cancer types: aberrant LCD-mediated interactions and condensate formation.

In neurodegenerative diseases, particularly amyotrophic lateral sclerosis and frontotemporal dementia, LCD-containing RNA-binding proteins such as TDP-43, FUS, and hnRNPA1/2 are implicated in pathogenesis. Under physiological conditions, these proteins form dynamic, reversible condensates that regulate RNA metabolism [73,74]; disease-associated mutations, however, accelerate their conversion into pathological solid aggregates, causing neurotoxicity [74–76]. PTMs further modulate this transition. For example, phosphomimetic substitutions at the TDP-43 C-terminus that mimic pathological Ser403/404 and Ser409/410 phosphorylation alter the electrostatic contribution to LLPS, favoring condensation at low ionic strength and conferring a biphasic dependence on salt concentration [77]. Notably, phosphorylation at Ser409/410 is a consistent feature of pathological TDP-43 inclusions across sporadic and familial TDP-43 proteinopathies [78].

LCD-mediated regulation also underlies developmental disorders. The LCD-containing regulator ABCF1 interacts with the pluripotency factor SOX2, and its loss disrupts transcription-genome surveillance coupling and stem cell homeostasis [79]. Polyalanine repeat expansions in HOXD13, which cause hereditary synpolydactyly, impair its ability to co-condense with Mediator. Rather than forming aggregates as classically described [80,81], expanded HOXD13 was shown more recently to form compositionally distinct condensates. These condensates reprogram HOXD13 target-gene transcription in a cell-type-specific manner, a phenomenon termed ‘condensate unblending.’ A similar effect has been observed for repeat expansions in RUNX2 associated with cleidocranial dysplasia [82]. In addition, disease-associated frameshift mutations that generate arginine-rich basic tails can cause aberrant nucleolar partitioning, as shown for HMGB1. From a genome-wide catalogue of more than 600 candidate frameshifts with similar charge-inverting potential, 12 of 13 tested disease-associated variants—spanning unrelated genes rather than HMGB1 itself—also caused nucleolar mispartitioning, suggesting that IDR charge inversion may represent a broadly relevant pathomechanism in rare genetic diseases [83].

These disease contexts share dysregulated LCD-mediated assembly behavior as a common theme, pointing to the aberrant assemblies as potential therapeutic targets. Oncogenic condensates are increasingly recognized as pharmacologically tractable. Emerging strategies to target them include small-molecule phase-separation modulators, peptide-based inhibitors, and targeted protein degradation [84]. As proof of concept, rationally optimizing inhibitors of androgen receptors based on the molecular determinants of their phase separation has yielded compounds with enhanced binding affinity and inhibitory potency, as well as antitumor activity in models of castration-resistant prostate cancer. These findings suggest that IDR-targeted drug discovery may represent a promising therapeutic strategy [85].

## Experimental approaches to study LCD-mediated transcriptional regulation

Understanding how LCDs regulate transcription often requires a combination of *in vitro* and live-cell approaches: *in vitro* assays define the biophysical rules governing LCD-mediated assembly, while live-cell methods capture the dynamics, selectivity, and regulatory consequences of these interactions in physiological contexts.

*In vitro* reconstitution assays, including droplet and hydrogel formation assays, probe the concentration dependence, reversibility, and material properties of LCD-mediated condensates under defined conditions [22,86]. Biochemical enrichment approaches such as the biotinylated isoxazole precipitation assay complement these by identifying phase-separation-prone proteins and their associated factors via mass spectrometry and transcriptome-wide analyses [86,87].

In living cells, fluorescence recovery after photobleaching (FRAP) provides a semi-quantitative measure of assembly dynamics and can help distinguish liquid-like condensates from gel- or solid-like aggregates [14,18]. Single-particle tracking complements FRAP by directly quantifying molecular diffusion coefficients, bound fractions, and residence times, making it particularly powerful for resolving heterogeneous molecular populations [88–90].

Optogenetic tools, such as OptoDroplets and Corelets, and chemogenetic tools, such as iPOLYMER, SPPIER, and SPARK, enable inducible, spatiotemporally controlled perturbation of LCD-mediated condensate formation. These approaches have been widely used to investigate how induced condensation relates to transcriptional activity [91–98].

The LacO array assay visualizes LCD–LCD interactions at a defined genomic locus by tethering a LacI-fused LCD of interest and quantifying the enrichment of a co-expressed, fluorescently tagged second LCD [9]. This assay has revealed several examples of sequence-encoded interaction selectivity: FET-family LCDs interact with one another but not with Sp1 [9]; NPM1c selectively recruits RNAP II CTD and the nuclear export protein XPO1 but not ENL or the scaffold protein Menin [69]. Beyond pairwise interaction readouts, the LacO array has also been combined with proximity labeling mass spectrometry to identify the interactome of a tethered LCD [99].

However, application of this approach is limited to the particular LacO-array-integrated cell line. Moreover, the system generates only a single condensate per nucleus, occupying a very small fraction of the nuclear volume and recruiting relatively few interaction partners of the target LCD, thereby limiting detection sensitivity by mass spectrometry. In addition, it cannot be used to characterize cytoplasmic condensates. To overcome these limitations, we recently developed phase-separation-induced interactome detection (PhaseID), which combines chemically inducible phase separation with proximity labeling mass spectrometry to identify the multivalent interactome of a given LCD in its condensates in live cells. This method is broadly applicable to LCDs with phase-separation propensity across diverse cell types. PhaseID revealed that transactivating LCDs each recruit a unique set of partners to activate transcription through distinct pathways and that proteins partitioning into specific condensates share sequence-encoded features that vary with the nucleating LCD [35].

A limitation across many studies is the reliance on overexpression systems, which can drive artifactual condensate formation and complicate interpretation, especially for data describing the functional relevance of condensates [100,101]. This limitation has started to be addressed by imaging endogenous proteins labeled through CRISPR-mediated genome editing [9,102,103]. Approaches such as proximity-assisted photoactivation, which studies IDRs at endogenous expression levels, will be useful for understanding the functional relevance of LCD-mediated assemblies [35,101,104]. Another limitation is related to the broad use of FRAP. Although dynamic molecular exchange measured by FRAP has often been used as evidence of LLPS in cells, FRAP recovery alone is increasingly recognized as insufficient to prove the LLPS nature of a compartment, as similar dynamics can reflect transient molecular interactions in the absence of LLPS [105]. A recently developed approach, model-free calibrated half-FRAP (MOCHA-FRAP), leverages preferential internal mixing within condensates formed by LLPS to distinguish bona fide LLPS from molecules binding to spatially clustered sites without undergoing LLPS [106]. Development of similar live-cell assays may help to rigorously diagnose LLPS in cells in the future.

## Perspectives

- **Importance of the field:** LCDs are highly prevalent within transcription regulators, which organize functional assemblies and enable the selective recruitment of transcriptional machinery to target genes, thereby facilitating gene activation. Dysregulation of these processes has been implicated in a broad spectrum of human diseases, underscoring the critical roles of LCD-mediated interactions in cellular function.
- **Current thinking:** Sequence features of LCDs, such as amino acid composition, patterning, and PTMs, often determine their ability to undergo multivalent interactions. Functional regulation of LCD-mediated assemblies can be achieved in various ways, including tuning local LCD concentration, PTMs, and RNA-mediated feedback. Productive transcriptional control has been found to require optimal levels of LCD–LCD interactions. Dysregulated LCD–LCD interactions can lead to aberrant transcription programs in disease. Despite the rapid progress in the field, many fundamental questions about LCD-mediated transcriptional control remain under active investigation.
- **Future directions:** Progress will benefit from integrating proteomic and single-molecule imaging approaches at endogenous expression levels with machine learning frameworks trained on high-quality experimental datasets. Together, these advances could move the field beyond descriptive cataloging toward predictive, sequence-based models of LCD function [23,107,108], ultimately enabling rational LCD design, the identification of disease-relevant interaction interfaces, and the development of therapies that selectively target pathological LCD interaction networks [85].

## Open Access

Open access for this article is provided via our Subscribe to Open programme, made possible through the support of our subscribing institutions. Learn more here: https://portlandpress.com/pages/open_access_options_and_prices#conditional.

## Abbreviations

CTD: C-terminal domain
FRAP: fluorescence recovery after photobleaching
HP1: heterochromatin protein 1
IDRs: intrinsically disordered regions
LCDs: low-complexity domains
LLPS: liquid–liquid phase separation
PhaseID: phase-separation-induced interactome detection
PTMs: post-translational modifications
RNAP II: RNA polymerase II
SLiMs: short linear motifs
TFs: transcription factors
